# Wearable Devices for Quantifying Atrial Fibrillation Burden: A Systematic Review and Bayesian Meta-Analysis

**DOI:** 10.3390/jcdd12040122

**Published:** 2025-03-30

**Authors:** Ioannis Anagnostopoulos, Dimitrios Vrachatis, Maria Kousta, Sotiria Giotaki, Dimitra Katsoulotou, Christos Karavasilis, Gerasimos Deftereos, Nikolaos Schizas, Dimitrios Avramides, Georgios Giannopoulos, Theodore G. Papaioannou, Spyridon Deftereos

**Affiliations:** 1 Department of Interventional Cardiology and Electrophysiology, Evgenidio Hospital, 11528 Athens, Greece; dvrachatis@gmail.com (D.V.);; 2 Cardiology Department, Athens General Hospital “G. Gennimatas”, 11527 Athens, Greece; 3 Department of Biomedical Engineering, Medical School, National and Kapodistrian University of Athens, 11527 Athens, Greece; teogpap@gmail.com; 4 2nd Department of Cardiology, National and Kapodistrian University of Athens, 11527 Athens, Greece; 5 Department of Cardiothoracic Surgery, Hygeia Hospital, 15123 Athens, Greece; nikschizas@gmail.com; 6 3rd Department of Cardiology, Aristotle University of Thessaloniki, 54124 Thessaloniki, Greece; ggiann@auth.gr

**Keywords:** atrial fibrillation, burden, prognosis, wearables, smartwatches

## Abstract

Background: Atrial fibrillation (AF) is the most common supraventricular arrhythmia and is associated with an impaired prognosis. Studies using implantable cardiac monitors suggest that this association is closely linked to AF burden, defined as the percentage of time spent in AF. Consequently, there is a growing need for affordable and comfortable alternative devices, such as wearables, capable of reliably monitoring AF burden in patients with AF. Methods: Major electronic databases were searched for studies comparing AF burden quantification using wearables and reference ECG monitoring methods. A Bayesian approach was adopted for the final analysis. Results: Six studies, including a total of 448 patients and 36,978 h of valid simultaneous recordings, were analyzed. Bayesian analysis revealed no statistically significant differences between wearables and reference methods in AF burden quantification. The mean error was 1% (95% CrIs: −4% to 7%). Similar findings were observed in the subgroup analysis of studies assessing only smartwatches. Between-study heterogeneity was low, and no evidence of publication bias was detected. Conclusion: Our analysis suggests that AF burden quantification using wearables is comparable to reference ECG monitoring methods. These findings support the potential role of wearables in clinical practice, particularly for research and prognostic purposes. However, more studies are needed to determine whether the observed statistical equivalence translates to clinical significance, thereby supporting the widespread use of wearables in the assessment of rhythm control therapeutic strategies.

## 1. Introduction

Atrial fibrillation (AF) is the most common supraventricular arrhythmia, affecting more than 59 million people globally as of 2019, with its prevalence expected to rise further due to population aging [1]. AF is associated with impaired prognosis. Beyond embolic stroke, it significantly impairs quality of life [2] and raises the likelihood of developing heart failure or the worsening of pre-existing heart failure [3,4]. Moreover, a growing body of evidence suggests that AF may be associated with increased all-cause mortality [5].

Continuous heart rhythm monitoring through implantable devices has demonstrated a strong, likely dose-dependent association between AF burden—defined as the percentage of time spent in AF—and these adverse outcomes [6]. Consequently, AF burden has not only provided valuable prognostic insights but has also emerged as a potential therapeutic target [7].

However, much of the available evidence comes from patients with implantable defibrillators or pacemakers, populations typically characterized by higher comorbidity. Extrapolating these findings to patients with fewer comorbidities may be problematic and misleading. To bridge this gap, alternative methods for reliable AF monitoring are needed to better understand its prognostic and therapeutic implications in a broader, less comorbid AF population.

Wearable devices offer an affordable and user-friendly solution, promoting continuous advancements in health data monitoring and tracking [8]. In a recent review article, Tedschi et al. summarized key insights into the evolving role of telemedicine in improving heart failure management. Within this framework, they highlighted promising evidence supporting the role of wearables in these patients [9]. Among their various applications, wearables have demonstrated diagnostic accuracy in correctly identifying AF episodes, mainly through photoplethysmography [10]. More recently, encouraging findings have emerged regarding their performance in quantifying AF burden [11], raising hopes for the integration of wearables into the tele-monitoring of patients with AF, which could potentially improve their long-term management.

In this systematic review and meta-analysis, we aim to synthesize and analyze the existing literature data, comparing AF burden quantification using wearable devices with reference ECG monitoring methods. We hope our findings will enhance the understanding of the role of wearables in clinical practice and pave the way for their integration into future research protocols.

## 2. Methods

This systematic review and meta-analysis was conducted in accordance with PRISMA guidelines [12]. The predefined protocol was registered in the PROSPERO database (616546).

### 2.1. Search Strategy

A systematic literature search was performed up to October 2024 in two databases: Medline (via PubMed) and Scopus. The following keyword-based strategy was used: ((wearable) OR (smartwatch) OR (wristband) OR (photoplethysmography)) AND (atrial fibrillation) AND (burden). Additionally, reference lists of included manuscripts were reviewed for potentially relevant articles (snowball strategy). No date restrictions were applied. Only articles available in English were included.

### 2.2. Study Selection

All articles identified in the initial search were screened by two independent reviewers (D.K. and C.K.) at the title and abstract levels. Studies deemed potentially eligible were then reviewed in full text to determine inclusion in the final analysis. Disagreements were resolved through consensus with an expert (G.G.). Studies eligible for final analysis included prospective or retrospective studies of adult patients—either with or without a history of AF—that compared the correlation of AF burden estimation between wearables and a reference ECG monitoring method. Both free-living and hospitalized patients were included, while no restriction was applied in terms of the technology/algorithms used in wearables’ software. The minimum study duration for inclusion was 24 h.

### 2.3. Data Extraction

Data of interest were extracted into a predesigned *Microsoft Office Excel 2007 form by two independent reviewers* (D.K. and C.K.) and then crosschecked for any disagreements. Disagreements were resolved by consensus with a senior (G.G.). In cases of inadequate data reporting (in otherwise eligible studies), corresponding authors were contacted electronically (via email).

### 2.4. Risk of Bias

The JBI critical appraisal checklist for diagnostic test accuracy studies [13] was used (in duplicate) to assess the risk of bias regarding the outcome of interest. Disagreements were resolved by consensus. Studies with an overall score ≥ 70% were considered of high quality, whereas those scoring < 50% were rated as low quality. Publication bias was evaluated using a Bayesian regression model based on the regression of the effect sizes against their standard errors, similarly to the classic Egger’s test [14].

### 2.5. Statistical Analysis

Continuous variables were summarized as the mean (standard deviation). When continuous data were reported as the median with an interquartile range, the median was assumed as the mean and the standard deviation was estimated by dividing the interquartile range by 1.35. AF burden was defined as the percentage of time spent in AF relative to the total monitoring time. To estimate the true difference between methods, we used the mean error along with its standard error as the outcome of interest. In cases where this outcome was not directly available in the text, Bland–Altman plots were used for visual estimation.

Given the limited number of eligible studies and the methodological differences—such as the use of different software across studies—a high degree of heterogeneity was expected. Since even small differences in AF burden may have clinical significance, it is crucial to estimate the probability that the true effect size lies within a specific range. Bayesian models are better suited for this task because they provide credible intervals, which directly reflect the probability distribution of the true effect size. In contrast, frequentist models are less reliable when dealing with small datasets and high heterogeneity, as they tend to underestimate uncertainty and produce artificially narrow confidence intervals, increasing the risk of overconfident conclusions. To address these issues, we adopted a Bayesian approach, which allows us to derive a posterior distribution and probabilistically estimate the true effect size. Given the anticipated degree of heterogeneity between studies, we adopted a hierarchical Bayesian random effects model [15]. This model accounts for the complexity of the available data, providing a more accurate estimate of the true effect size. We chose weakly informative priors. For the intercept, we assumed a normal distribution with a mean around zero, leaving enough uncertainty by setting the standard deviation to 0.45 (normal (0, 0.45)). To account for the expected between-study heterogeneity, we selected a heavy-tailed half-Cauchy distribution with a scale of 1 (half-Cauchy (0,1)). The Markov chain Monte Carlo algorithm (with a target Rhat value < 1.1) was employed to obtain the posterior distribution and the 95% credible intervals (CrIs) [16]. Finally, we calculated the Bayes factor to evaluate the null hypothesis that there is no difference between the methods in quantifying AF burden. Values > 3 strongly support the alternative hypothesis, and values < 1/3 strongly support the null hypothesis, while values between 1/3 and 3 do not allow for robust conclusions [17]. Additionally, sub-analysis was performed to investigate the effect size in studies using only smartwatches, while meta-regression analyses were conducted to investigate the influence of other confounders (mean age, sex, hours of monitoring). All analyses were performed using R Foundation software (version 4.1.2) with the brms package.

## 3. Results

**Figure 1:**
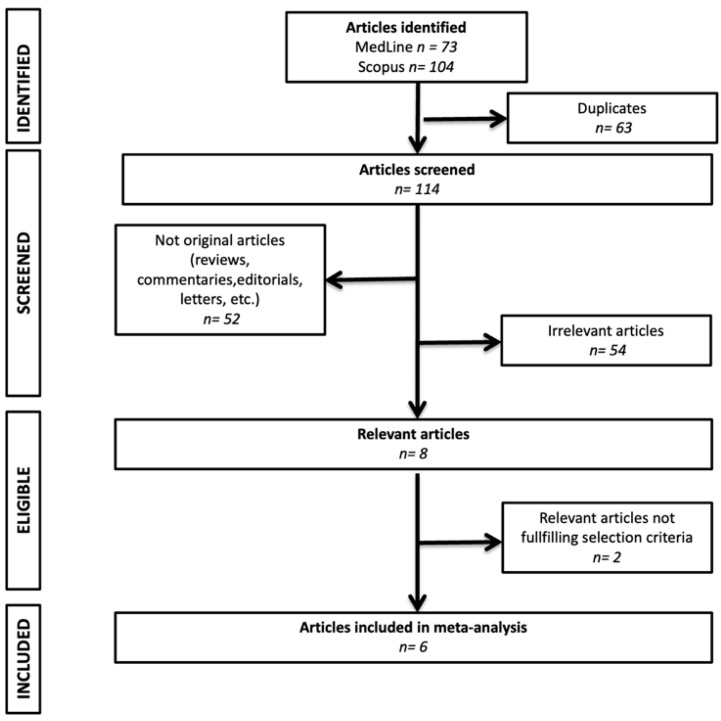
Flow of study selection.

The initial search identified 112 articles, of which 7 were eligible for the final analysis. One was excluded due to duplication, thus leaving six studies in the final analysis [18,19,20,21,22,23]. The flow of study selection is depicted in Figure 1. The included studies involved 778 patients (54% males) with a mean (SD) age of 66 [12] years, of whom 90% had a history of AF (81.5% paroxysmal). Five studies assessed smartwatches using photoplethysmography, while one study evaluated a wearable heart belt. As a reference method, four studies used an ECG patch and two studies used a Holter monitor. Most recordings were conducted in free-living settings. The proportion of non-interpretable recordings ranged from 0% to 50.7%. In total, 125,482 h of interpretable simultaneous recordings were analyzed across the parent studies to evaluate the diagnostic performance of wearable devices in AF detection. Almost 36,978 h of valid simultaneous recordings—from 448 patients—were analyzed to estimate the performance of wearable devices in quantifying AF burden. To calculate the mean error, all but one study focused exclusively on patients with documented AF episodes during the study period. The characteristics of the included studies are summarized in Table 1.

### 3.1. Quality Assessment

Through the use of the JBI critical appraisal checklist for diagnostic test accuracy studies, all studies were assessed as being of high quality (Table 2). In the regression analysis of the effect sizes against their standard errors, no significant publication bias was observed (Egger’s test modified: 0.27 (95%CrI: −0.87 to 1.54)).

### 3.2. Meta-Analysis Results

**Figure 2:**
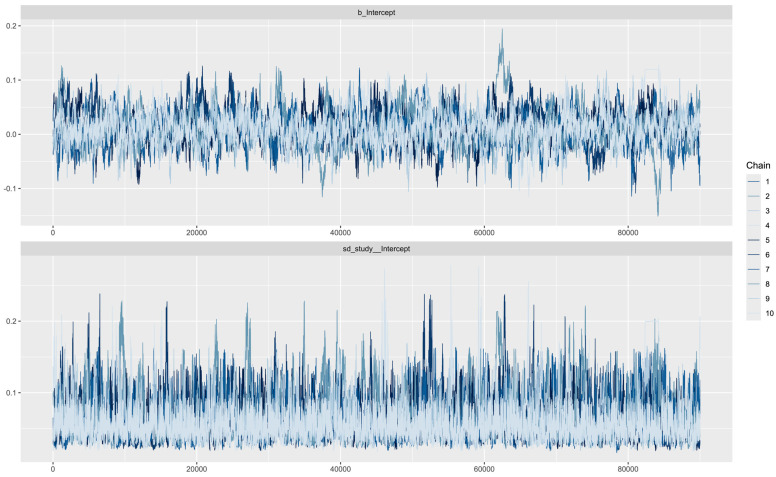
Trace plot illustrating model convergence for the mean error (upper diagram) and between-study heterogeneity (lower diagram). All studies are included in this analysis.

**Figure 3:**
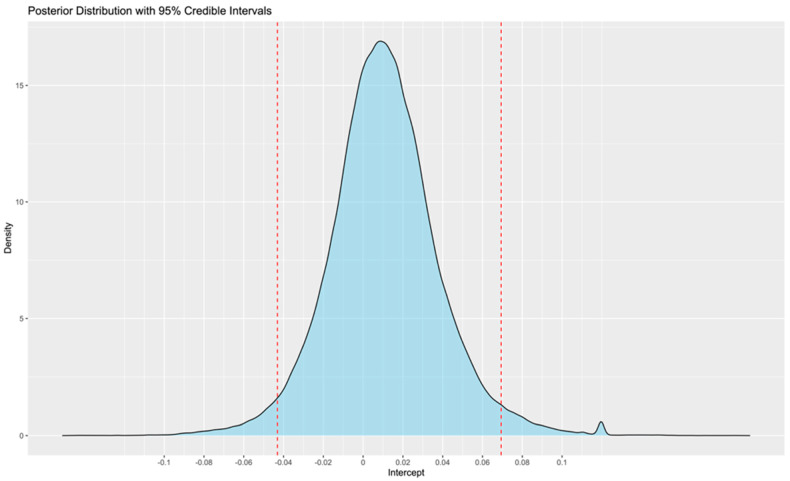
Posterior probability distribution of the mean error between wearables and reference methods. The red vertical lines denote the 95% credible intervals.

A Bayesian model using 10 chains with 100,000 iterations (the first 10,000 as a warm-up) achieved the desired convergence. The corresponding trace plots showed minor fluctuation around the mean, indicating credible meta-analysis results (Figure 2). The Bayesian meta-analysis documented no statistically significant differences between the two methods (mean error: 0.1; 95%CrIs: −0.04 to 0.07; Rhat: 1.01). The posterior probability distribution is displayed in Figure 3. Although between-study heterogeneity was limited, it was not negligible (sd: 0.06; 95%CrIs: 0.03 to 0.13; Rhat: 1.01). The Bayes factor was calculated to be 0.058, providing strong support for the null hypothesis.

Meta-regression analyses revealed no significant confounding effects from mean age, sex distribution, or monitoring duration.

**Figure 4:**
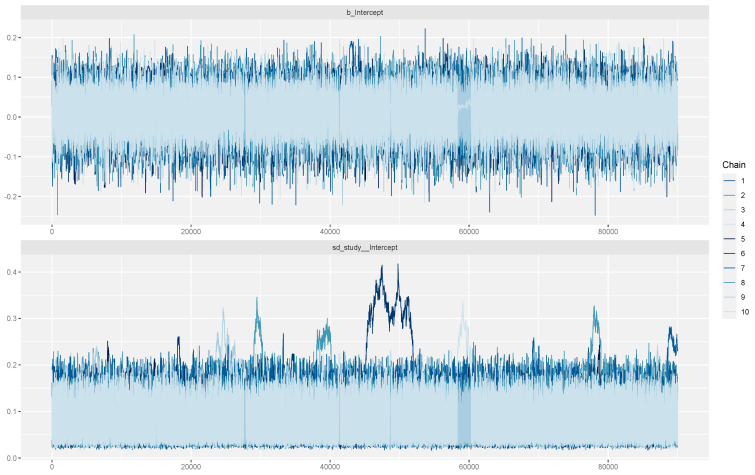
Trace plot illustrating model convergence for the mean error (upper diagram) and between-study heterogeneity (lower diagram). Only studies that examined smartwatches are included in this analysis.

**Figure 5:**
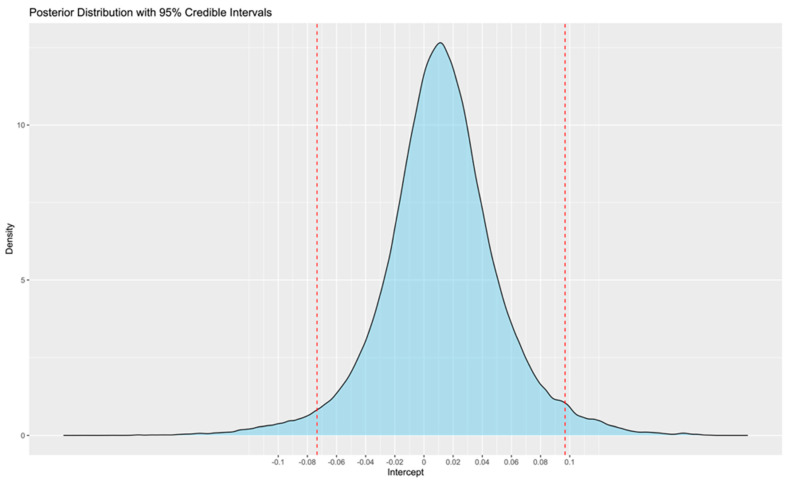
Posterior probability distribution of the mean error between wearables and reference methods, limited to wearables using PPG. The red vertical lines denote the 95% credible intervals.

A sub-analysis including the five studies that exclusively assessed smartwatches was also performed (375 patients, 37,754 h of simultaneous recordings). The trace plots again confirmed credible convergence (Figure 4). According to the Bayesian meta-analysis, no statistically significant difference was identified (mean error: 0.01; 95%CrIs: −0.07 to 0.10; Rhat: 1). The posterior probability distribution is illustrated in Figure 5. Limited between-study heterogeneity was observed (sd: 0.08; 95%CrIs: 0.03 to 0.21; Rhat: 1.01). The Bayes factor was 0.076, which strongly supports the null hypothesis.

## 4. Discussion

In this systematic review and meta-analysis, we evaluated the ability of wearable devices to quantify AF burden by analyzing data from studies that directly compared wearables to reference ECG monitoring methods. All included studies were prospective and of high methodological quality, while no publication bias was identified. Most of them focused on smartwatches utilizing photoplethysmography technology. Given the limited number of studies and patients, we adopted a Bayesian approach to allow for a probabilistic estimation of the true effect size. The findings of our analysis strongly support the null hypothesis, indicating no statistically significant difference between wearables and reference ECG methods. The mean error was estimated to be +1%, with a 95% CrI ranging from −4% to 7%. No confounding effects related to mean age, sex, or monitoring duration were identified. In a sub-analysis, limited to smartwatch studies, the mean error fell within a slightly broader scale (from −7% to 10%). However, it is important to note that these findings are applicable only to valid recordings obtained during simultaneous monitoring. Given the high proportion of non-interpretable recordings in most studies and the potential for gaps when devices are not worn, caution is warranted when interpreting periods without monitoring.

Previous research has established the high diagnostic accuracy of wearable devices for AF detection, with no significant difference between single-lead ECG and photoplethysmography-based devices [24,25]. However, to our knowledge, this is the first meta-analysis to focus on the quantification of AF burden, providing promising results that could be relevant for both clinical practice and future research.

Clinical AF diagnosis requires the recording of a 12-lead ECG demonstrating an irregular rhythm without visible P waves. AF is further classified as paroxysmal, persistent, or permanent depending on the duration of the arrhythmia and whether efforts to restore normal sinus rhythm have been abandoned [26]. This classification holds clinical significance, as previous studies indicate that the progression from paroxysmal to persistent form is associated with worse outcomes [27]. However, treating AF solely as a categorical variable may be misleading. Emerging evidence suggests that AF burden—the percentage of time a patient is in AF—provides additional prognostic value. Findings from the SOS AF Project indicate that for patients with cardiac implantable devices, each additional hour of AF burden corresponds to a 3% increase in the risk of stroke [28]. Similarly, Steinberg et al. reported that every 10% increase in AF burden was associated with a 9% higher risk of new-onset heart failure and a 5% increase in all-cause mortality [29]. Notably, the CIRCA-DOSE study demonstrated that even AF burdens exceeding 0.1% were associated with increased healthcare utilization [30].

The clinical value of AF burden is further supported by recent studies demonstrating that its reduction is associated with prognostic benefits. In a sub-analysis of the CASTLE-AF Trial, Brachmann et al. reported that reducing AF burden below 50% significantly decreased the incidence of hard clinical outcomes [31]. These benefits also extend to quality-of-life improvements [32,33]. Thus, beyond its general prognostic significance, AF burden has emerged as a potential therapeutic target, particularly following rhythm control interventions [34].

Most available evidence on AF burden quantification comes from studies using implantable devices, making extrapolation to a healthier population potentially misleading. Insertable cardiac monitors have demonstrated high accuracy in both detecting AF episodes and quantifying AF burden [35], rendering them the most precise option for AF monitoring. However, the implantation process as well as the high cost and the limited longevity currently restricts their widespread use, reserving them primarily for patients where an accurate and timely diagnosis is mandatory [36,37]. This underscores an unmet need for low-cost, user-friendly, and accurate alternatives for AF monitoring.

Technological advancements have led to the increased adoption of wearable devices, which are now commonly used to promote a healthy lifestyle and manage existing conditions [38]. In cardiology, wearables have been used to monitor parameters such as heart rhythm using ECG and photoplethysmography, a technology that detects blood volume changes in the microvascular bed [39]. Recent studies have demonstrated the accuracy of these devices in correctly identifying AF episodes [25]. Given that wearables are typically worn throughout the day, there is a growing interest in their potential to reliably monitor and quantify AF burden.

In this systematic review, we gathered the existing literature data to evaluate the ability of wearables to quantify AF burden. Our analysis strongly supports the conclusion that there is no statistically significant difference between wearables and reference ECG methods in quantifying AF burden. Wearables tended to overestimate AF burden by 1%; however, the true difference likely lies between −5% and 7%, with 95% probabilities. This difference may be slightly higher for smartwatches using photoplethysmography. These findings are promising, supporting further evaluation of wearables in research protocols to better establish their reliability. They also pave the way for their use in clinical practice. Wearables could serve two primary purposes: first, for prognostic purposes, to identify patients at higher risk of adverse outcomes who may benefit from more aggressive rhythm control strategies; and second, to optimize therapeutic strategies, using AF burden reduction as a treatment target. However, these findings should be interpreted with caution. The clinical significance of different AF burden levels and the optimal rate of reduction remain uncertain. Consequently, we cannot yet determine whether differences, such as those of the observed magnitude, are truly clinically insignificant. Moreover, the high percentage of invalid recordings, coupled with periods when devices are not worn, may create substantial gaps in AF monitoring.

This meta-analysis also has several limitations. Most studies used different devices and algorithms to quantify AF burden, which may be a potential source of heterogeneity. Wearable device recordings also had a high percentage of non-interpretable data, potentially concealing valuable information. These recording limitations could arise from device-related factors (e.g., sensor quality, algorithm performance) or patient-related factors (e.g., movement artifacts, skin characteristics). Additionally, the available data did not allow us to assess potential confounders such as body mass index and skin color, preventing the identification of patient phenotypes that may be prone to outlier measurements. Finally, the current data do not allow us to definitively conclude whether this lack of statistical significance reflects a true absence of clinically meaningful differences.

## 5. Conclusions

In conclusion, this systematic review and meta-analysis evaluated the performance of wearable devices in quantifying AF burden. We included studies comparing wearables to reference ECG monitoring methods, focusing on the mean error in burden estimation. Most studies assessed smartwatches utilizing photoplethysmography to quantify AF burden in free-living settings. Bayesian analysis indicated no statistically significant difference between the methods, with the true difference estimated to range between −4% and +7%. These results are encouraging, supporting the integration of wearables into clinical practice, particularly for prognostic and research purposes. However, they simultaneously highlight that further research is necessary to confirm the reliability of wearables for therapeutic monitoring and treatment response assessment. Future efforts should also focus on optimizing algorithms to reduce non-interpretable data and improve the accuracy of these devices.

## 6. Clinical Perspectives

- Wearable devices, at least theoretically, could be used in a free-living setting to provide useful information regarding AF burden;
- According to the meta-analysis of the existing literature, no statistically significant difference was observed between wearables and reference ECG monitoring methods, with the mean error estimated at 1% and the 95% CrIS ranging from −4 to 7%;
- This range may be slightly larger for smartwatches;
- These findings support the potential role of wearables in future research and clinical practice. However, the relationship between different levels of burden and outcomes is not yet clear. Therefore, further research is needed to determine whether differences, such as those of the observed magnitude, are clinically significant or not.

## Figures and Tables

**Table 1:** Summary of studies’ characteristics.

Study	N	Age (y)	Males (%)	PAF (%)	PeAF (%)	FU (d)	Wearable	Noise (%)	Simultaneous Recording (h)	Recording Setting
Santala OE et al., 2021 [18]	73	77 (10)	52.1	n/a	n/a	1	Heart Belt	19.6	1224	Hospital
Zhang H et al., 2021 [19]	53	66.3 (11.8)	50.9	9.4	18.9	28	Smartwatch	0	3812	Free living
Zhu L et al., 2022 [20]	204	62.6 (11.6)	73	77.9	7.8	28	Smartwatch	32.3	20,700	Free living
Reissenberger P et al., 2023 [21]	92	73.3 (10.4)	n/a	100	0	2	Smartwatch	50.7	547	Free living/Hospital
Poh MZ et al., 2023 [22]	111	65 (11)	55	100	0	14	Smartwatch	22.8	7667	Free living
Zhao Z et al., 2024 [23]	245	63.1 (10.8)	39.2	62.5	37.6	2	Smartwatch	37.2	3028	Hospital

d: days; FU: follow-up; h: hours; N: number of patients in parent studies; PAF: paroxysmal atrial fibrillation; PeAF: persistent atrial fibrillation; y: years; N/a: not available.

**Table 2:** Quality assessment of the included studies.

Study	1	2	3	4	5	6	7	8	9	10	Overall
Santala OE et al., 2021 [18]	yes	yes	yes	yes	n/a	Yes	yes	n/a	yes	no	87.50%
Zhang H et al., 2021 [19]	yes	yes	yes	yes	n/a	Yes	yes	n/a	yes	no	87.50%
Zhu L et al., 2022 [20]	yes	yes	yes	yes	n/a	Yes	yes	n/a	yes	no	87.50%
Reissenberger P et al., 2023 [21]	yes	Yes	yes	yes	n/a	Yes	yes	n/a	yes	no	87.50%
Poh MZ et al., 2023 [22]	yes	Yes	yes	yes	n/a	Yes	yes	n/a	yes	no	87.50%
Zhao Z et al., 2024 [23]	yes	Yes	yes	yes	n/a	Yes	yes	n/a	yes	yes	100%

N/a: not available.
